# Experience of use of bedside ultrasonography for detection of Abdominal aortic aneurysm in a tertiary medical center in Taiwan, ROC

**DOI:** 10.1186/2036-7902-7-S1-A31

**Published:** 2015-03-09

**Authors:** Ying-Lin Tan, Kuo-Chih Chen, Tzong-Luen Wang

**Affiliations:** Emergency Department, Shin Kong Wu Ho-Su Memorial Hospital, ROC, Taipei, Taiwan

**Keywords:** Emergency Department, Aortic Aneurysm, Abdominal Aortic Aneurysm, Aortic Dissection, Abdominal Aortic Aneurysm

## Background

Rupture of abdominal aortic aneurysm is one of the life-threatening diseases in Emergency Department (ED) and it can be misdiagnosed. It happens mostly in the people who are in average of age 75 to 84 years.Use of bedside ultrasonography has been became more common in daily practice of emergency physician (EP) for approaching patient in ED, which has chief complaint of acute abdomen.Especially in the life-threatening rupture abdominal aortic aneurysm, bedside sonography can be more safe and adequate time-saving tool for EP for detection, diagnosis and next crucial management. We reviewed the database of an tertiary medical center for collection of first diagnosis of abdominal aortic aneurysm made in Emergency Department.

## Objective

To acknowledge the utility of bedside ultrasonography for detection of AAA in ED.

## Patients and methods

SKH hospital is a tertiary medical center in Taipei. There have average 5000 to 6000 people visit to ED in each month. We searched the patient who visited to Emergency Department with diagnosis of abdominal aortic aneurysm (AAA) from 2010 year through April of 2014via patient medical information database of our hospital.

Total 77 patients were included, 18 patients was excluded due to history of AAA,23 patients was excluded due to no diagnosis of AAAbut with alternative diagnosis such as thoracic aneurysm, aortic dissection and so on. Total 31 patients were first diagnosed of AAA in ED within these 4 years and 4 months. Almost two of third of these cases was first diagnosed by utility of bedside ultrasonography. Table 1.

**Table 1:** Collection of patient which visited to ED with diagnosis of AAA

Year	Total number	History of AAA	No diagnosis of AAA	First diagnosis of AAA	Detection First by bedside sonography
2010/1/1~12/31	11	3	4	4	3
2011/1/1~12/31	23	4	9	10	9
2012/1/1~12/31	18	7	4	6	6
2013/1/1~12/31	21	6	7	8	4
2014/1/1~4/30	4	0	1	3	2
Total	77	18	23	31	24

## Result

Almost the emergent cases were diagnosed of ruptured AAA within 10 to 20 minutes by bedside ultrasonography.Utility of bedside ultrasonography shortened the time of Computed topography (CT) imaging study and decision making to crucial management (Surgical repair)especially in overcrowded ED. These cases should be diagnosed rapidly and treated aggressivelyotherwise they were dead. Bedside sonography has been also played a role in diagnosing the AAA which has atypical presentation such as hematuria or scrotal pain in our experience.

The larger size of AAA the easier to detect by bedside ultrasonography. Detection of rupture of AAA by bedside ultrasonography was amazingly noted in our experience ( 6 of 8 ruptured cases). In addition, use of ultrasonography in patient who is suspected AAA make EP shorten the time to computed topography performed in overcrowded ED and surgical repair if it was ruptured.

In rare condition like mycotic aneurysm, utility of bedside ultrasonography sometimes plays a role, we have one case of mycotic aneurysm first detected by ultrasonography.

## Conclusion

Bedside ultrasonography became a convenient and diagnostic tool for detection of AAA in

Emergency Department even its dangerous complication like rupture or getting infected.

**Figure 1:**
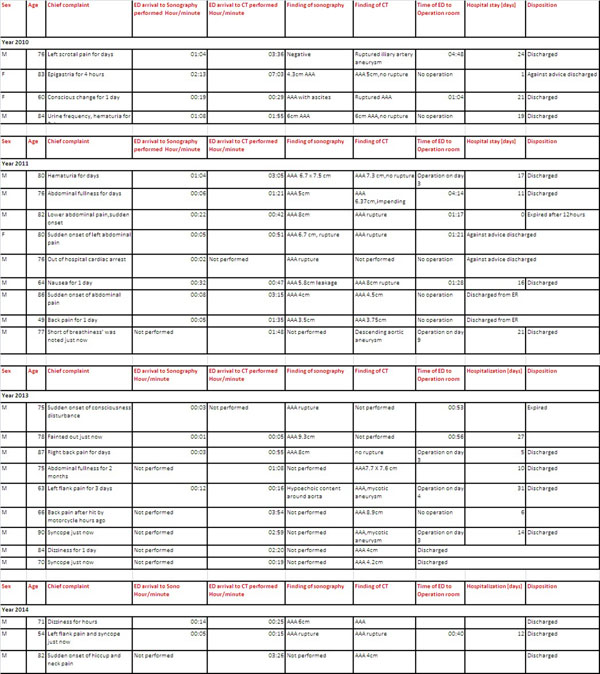
Data of patients which were diagnosed as Abdominal Aortic Aneurysm in our Emergent Department within 2010/1/1 to 2014/4/30

